# A Comprehensive Evaluation of the Attenuation Characteristics of Some Sliding Bearing Alloys under 0.015–15 MeV Gamma-Ray Exposure

**DOI:** 10.3390/ma15072464

**Published:** 2022-03-27

**Authors:** Merfat Algethami, Awad A. Ibraheem, Shams A. M. Issa, Huseyin O. Tekin, Antoaneta Ene, Maria Pyshkina, Mohamed Rashad, Ghada ALMisned, Hesham M. H. Zakaly

**Affiliations:** 1 Physics Department, Faculty of Science, Taif University, P.O. Box 11099, Taif 21944, Saudi Arabia; m.algethami@tu.edu.au; 2 Physics Department, King Khalid University, P.O.Box 9004, Abha 61421, Saudi Arabia; awibrahem@kku.edu.sa; 3 Physics Department, Faculty of Science, Al-Azhar University, Assiut 71524, Egypt; shams_issa@yahoo.com; 4 Physics Department, Faculty of Science, University of Tabuk, Tabuk 71451, Saudi Arabia; mra77_6@yahoo.com; 5 Medical Diagnostic Imaging Department, College of Health Sciences, University of Sharjah, Sharjah 27272, United Arab Emirates; tekin765@gmail.com; 6 Computer Engineering Department, Faculty of Engineering and Natural Sciences, Istinye University, Istanbul 34396, Turkey; 7 INPOLDE Research Center, Department of Chemistry, Physics and Environment, Faculty of Sciences and Environment, Dunarea de Jos University of Galati, 47 Domneasca Street, 800008 Galati, Romania; 8 Institute of Physics and Technology, Ural Federal University, 620002 Yekaterinburg, Russia; maria1pyshkina@gmail.com; 9 Department of Physics, Faculty of Science, Assiut University, Assiut 71524, Egypt; 10 Department of Physics, College of Science, Princess Nourah Bint Abdulrahman University, P.O. Box 84428, Riyadh 11671, Saudi Arabia; gaalmisned@pnu.edu.sa

**Keywords:** alloys, MicroShield-7.01, gamma-ray, EBF, EABF, dose rate

## Abstract

In this study, three different sliding bearing alloy samples were investigated in terms of their performance on attenuation characteristics and behavioral attitudes under 0.015–15 MeV gamma-ray exposure. Accordingly, different types of advanced calculation methods were utilized to calculate the radiation shielding parameters. Next, several gamma-ray shielding parameters and exposure rates in addition to fast neutron removal cross-section were determined. Furthermore, exposure and energy absorption buildup factors were determined by using G-P fitting method. Mass attenuation coefficients (MAC) values were recorded as 2.5246, 2.5703, and 2.5827 (cm^2^/g) for Alloy1, Alloy2, and Alloy3 samples at 15 MeV photon energy, respectively. At 40 mfp, the highest EBF values were reported as 1,376,274, 1,003,593, and 969,373 for Alloy1, Alloy2, and Alloy3 samples. The results of this extended investigation showed that the Alloy3 sample with the highest Pb reinforcement amount has superior shielding capability among the investigated samples. It can be concluded from the results that substitution of Pb with Bi in the recent alloy structure has a monotonic effect on different types of shielding parameters. Therefore, it can also be concluded that Pb is a remarkable tool for the improvement of the shielding properties of studied alloy structures.

## 1. Introduction

The extraction of new or man-made radioactivity and ionizing radiations triggered revolutions in science and technology around the globe. This upheaval altered our perception of the cosmos and impacted human quality of life. Nowadays, several artificial radiation sources are used at various institutions located across the globe for a variety of peaceful purposes. In most cases, ionizing radiation is used in diagnostic and therapeutic procedures in medicine [1]. In radiation medicine, radioisotopes generating gamma and radiopharmaceuticals are combined with a gamma camera to diagnose illness.

Additionally, oncology and therapeutic nuclear medicine are two therapeutic procedures that use ionizing radiation from a variety of radioactive isotopes and generators to treat cancer. Nuclear energy is also well-known for its usage in electricity production through nuclear reactors [2]. Ionizing radiation is used in research reactors to produce radioisotopes and characterize materials. Similarly, as seen by the expanding number of such facilities, the utilization of electron beams and gamma radiation for waste and water treatment has been recognized [3,4]. Regrettably, ionizing radiation has the potential to change the chemical composition’s physical and biological properties and to destroy non-biological components of living tissues. As a consequence, radiation protection has become an inherent part of all ionizing radiation quality assurance and control methods. A radiation shield is a shielding structure that is often used in all types of radiation facilities to protect people and their surroundings from the damaging effects of ionizing radiation [5,6,7]. While classic shielding materials such as concrete, lead, and depleted uranium continue to be employed, the increasing usage of ionizing radiation, environmental concerns, toxicity, endurance, space constraints, and cost constraints have motivated the quest for more acceptable shielding materials. Among them, several specific alloys used in next-generation shields have been thoroughly examined for their gamma-ray and neutron attenuation capabilities [8,9,10]. The term “alloy material” refers to a substance composed of many distinct kinds of metals. However, it may also be a mixture of a metal and a non-metallic element.

In recent years, alloys have gained increasing attention as other possible shielding materials. Alloys are metallic substances composed of multiple elements. By creating alloys, the properties of the original metals are enhanced including characteristics, such as strength, heat resistance, and shielding ability. Alloys can be classified based on their size, application, or base metal. A multitude of alloys with different base metals has been investigated for their radiation shielding ability. These include nickel, lead, tungsten, copper, iron, and more. Many of these base metals have shown great ability at attenuating radiation, making them viable materials for protection against the adverse effects of ionizing radiation [11,12,13,14,15,16]. Due to their excellent heat resistance properties, alloys are used predominantly in a wide range of applications, from aircraft and automobiles to electronics and batteries. Metal atom vibration due to the interaction with the radiation could lead to a reduction in the ductility of the material. As a result, the breaking of the ionic and covalent bonds takes place between the atoms in the materials. This can lead to a change in the material crystal structure, which results in permanent damage to the shielding equipment/materials. Materials used for the purpose of radiation shielding in a reactor environment must have high radiation absorption capacity, a combination of strength and metallurgical stability, and have high resistivity against temperature and chemical corrosion [17,18,19]. Radiation sensitivity presented a risk to the operators’ and patients’ health. As a result, shielding is recommended to limit exposure to ionizing radiation. The usual materials used for nuclear shielding are thought to be mostly lead (Pb) and lead-based compounds. However, international organizations, such as the International Atomic Energy Agency and the World Health Organization, have spurred academics on to develop new generation shields in their place during the past several years [20,21]. Several research investigations have explored the effect of different types and amounts of additives used in alloys for X-ray and gamma radiation shielding [22,23]. As Jamila S. Alzahrani et al. [22] investigated how four compositions of Al-alloys doped with different weights of Pb are synthesized and studied for their physical features and nuclear radiation shielding characteristics. Their study recommended that the production of alloys doped with heavy metal is demonstrated to be a successful technique to increase the shielding ability against ionizing radiation.

Additionally, providing this mixture enables the creation and enhancement of certain material characteristics. A critical material attribute worth researching is the ability of the material to protect nuclear radiation for healthcare, commercial, and personal purposes. The absolute composition of an alloy material may be altered to satisfy the needs of certain applications. Consequently, some extra materials may be included into the alloy composition to alter its qualities. The literature notion prompted us to conduct this work in order to ascertain the gamma-ray and other dosage attenuation properties of certain alloy shields. Accordingly, amorphous alloys containing Bi and Pb were studied for their potential use as nuclear shielding against ionizing radiation. We effectively evaluated the gamma shielding properties of the examined alloys using the MicroShield-7.01 [24,25,26] and Phy-X/PSD [27] platforms. Additionally, we used theoretical simulations to determine the alloys’ neutron radiation shielding properties. Recent analysis findings will improve the understanding of alloy shields’ behavioral responses to gamma-ray exposure.

## 2. Materials and Methods

In a present study, the alloy samples under investigation were produced by melting the mixture of raw materials, namely, aluminum, tin, bismuth, and lead (99.996 wt%). Copper and silicon were introduced into the alloys in terms of aluminum-10 wt% copper and aluminum-20 wt% silicon master alloys, respectively [28]. The MicroShield-7.01 software package is employed to calculate the radiation shielding parameters [29]. MicroShield uses built-in properties of individual materials to calculate the exposure rate with and without buildup factor. It is generally utilized for shielding design and source strength assessment from radiation measurements and is approved by the US NRC for different shielding studies. In this study, three different alloy samples were investigated in terms of their performance in gamma-ray shielding utilizations, as shown in Table 1. In particular, how Pb and Bi affect the attenuation properties of different alloys with nominal compositions (weight percentage) Al_82_Sn_15_Si_2.2_Cu_0.8_ Al_81.46_Sn_13_._1_Bi_2.44_Si_2.2_Cu_0.8_ and Al_81.02_Sn_13.17_Pb_2.81_Si_2.2_Cu_0.8_ were extensively reported; successfully investigated by Wang et al. [28] In their study.

The total photon interaction cross-section (*σ_t_*) of the samples was calculated with the help of the *µ_m_* according to the following equation [30,31]:

(1) $$\sigma_{t}=\frac{M\mu_{m}}{N_{A}}$$

where $M=\sum_{i}A_{i}n_{i}$ is the molecular weight of the sample, *A_i_* is the atomic weight of the *i*-th element, *n_i_* is the number of the formula units of a molecule, and *N_A_* is the Avogadro’s number. Effective atomic cross-section, *σ_a_*, was calculated using the following equation [32]:

(2) $$\sigma_{a}=\frac{\sigma_{t}}{\sum_{i}n_{i}}$$

Total electronic cross-section, *σ_e_*, was calculated by:

(3) $$\sigma_{e}=\frac{1}{N_{A}}\underset{i}{\sum }\frac{f_{i}A_{i}}{Z_{i}}{\left(\mu_{m}\right)}_{i}$$

where *f_i_* indicates the fractional abundance of the element *i* and *Z_i_* is the atomic number of the constituent element. The effective atomic numbers (*Z_eff_*) are related to *σ_a_* and *σ_e_* through the following equation [32,33]:

(4) $$Z_{eff}=\frac{\sigma_{a}}{\sigma_{e}}$$

As a single element has atomic number *Z*, the compound materials have an equivalent atomic number (*Z_eq_*) which describes the properties of glass systems. Because the gamma-rays’ partial interaction process with material depends on the energy, thus *Z_eq_* is an energy-dependent parameter. Using the WinXCom program [34], the total mass attenuation coefficient of selected glass samples and incoherent Compton scattering for elements from *Z* = 11 to 29 was obtained in the energy range 0.015–15 MeV. The *Z_eq_* was calculated by matching the ratio of incoherent Compton scattering to the *μ_m_* of selected glass samples with the identical ratio of a single element of the same energy. The following formula was used to interpolate the *Z_eq_* [35,36].

(5) $$Z_{eq}=\frac{Z_{1}\left(logR_{2}-logR\right)+Z_{2}\left(logR-logR_{1}\right)}{logR_{2}-logR_{1}}$$

where *Z*_1_ and *Z*_2_ are the elements’ atomic numbers identical to the ratios *R*_1_ and *R*_2_, respectively. *R* is the ratio of incoherent Compton scattering to the mass attenuation coefficient of the glass samples at a particular energy.

The G-P fitting parameters for the elements were taken from the report by the American Nuclear Society [37]. The G-P fitting parameters for the glass samples were logarithmically interpolated utilizing the same equation as follows [38]:

(6) $$C=\frac{C_{1}\left(logZ_{2}-logZ_{eq}\right)+C_{2}\left(logZ_{eq}-logZ_{1}\right)}{logZ_{2}-logZ_{1}}$$

where the two constants *C*_1_ and *C*_2_ correspond to *Z*_1_ and *Z*_2_, respectively. The fitting of G-P parameters was utilized to compute the EBF of glass samples as follows:

(7) $$B\left(E,\;X\right)=1+\frac{b-1}{K-1}\left(K^{x}-1\right)\;\;\;\;\;\;\;\;\;\;\;\;\;\;\;\;\;\;\;\;\;\;\;\;\;\;\;\;\;for\;K\neq 1$$

(8) B(K, X)=1+(b−1)x                                          for K=1

where

(9) $$K\left(E,\;x\right)=cx^{a}+d\frac{tanh\left(\frac{x}{X_{K}}-2\right)-tanh\left(-2\right)}{1-tanh\left(-2\right)}\;\;\;\;\;\;\;\;\;\;\;\;\;for\;\;x\leq 40$$

where *E* is the photon energy incident on the material, *X_K_* is the G-P fitting parameters, and *x* is the penetration depth in mfp. The variance of parameter *K* with *x* gives the dose photon multiplication and a variation in the shape of the spectrum.

## 3. Results and Discussions

The impact of bismuth and lead on radiation shielding on Al–Sn-based alloys has been studied. Their results showed that Bi reinforcement significantly increased the volume hardness of the Sn-rich phase by the consolidation of the solution, but Pb has only a small effect. Following recommendations of the IAEA and WHO, the importance of alternative shielding materials, which can be used in ionizing radiation facilities, is increasing day by day [9,10,39]. Therefore, this study was planned as a continuation to investigate the relationship between these interesting results and radiation attenuation of alloy samples, such as ASSC, ASBSC, and ASSPC.

**Figure 1:**
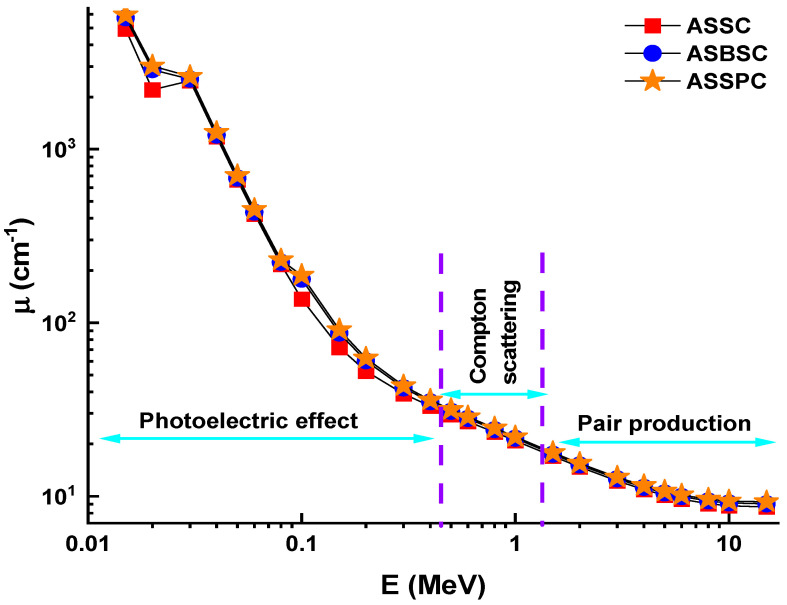
Linear attenuation coefficient (µ) for the 3 types of alloys.

As a first step, linear attenuation coefficients (LAC) of alloy samples were determined in 0.015–15 MeV photon energy range. The term LAC (µ) is known as an important shielding parameter for any type of candidate material that might be utilized in radiation facilities. This density-dependent parameter is a useful term to evaluate the performance of shielding material on attenuation of primary gamma rays. Figure 1 shows the variation of LAC values of ASSC, ASBSC, and ASSPC samples against photon energy. As clearly seen from Figure 1, variation of LAC values was directly affected by three basic interaction types, namely, photoelectric effect, Compton scattering, and pair production in different energy zones [40,41,42,43]. This situation is the nature of radiation interaction with matter depending on energy. In the low-energy region, LAC values are dramatically decreased due to the dominancy of the photoelectric effect. In the mid-energy region, Compton scattering was the dominant process that affected LAC values’ variation trend from sharp to smooth. However, the highest LAC values were reported for the ASSPC sample, which has the highest Pb concentration in its composition. To make it more straightforward, LAC values are listed as 8.668, 9.044, and 9.275 (cm^−1^) for ASSC, ASBSC, and ASSPC samples at 15 MeV photon energy, respectively. Mass attenuation coefficients (MAC) are defined as the density-independent material coefficient. MAC can be obtained for specific photon energy via dividing LAC values by sample density. The same trend is also evident for the variation of MAC versus photon energy (E) for studied alloy samples. The energy (gamma photon incident) and alloy structure (chemical composition) have modified the variation of MAC values. The gap of the MAC with energy is characteristic in three different zones. In the low energy area where the photoelectric effect is a dominant mechanism in the interaction, the MAC values decrease quickly as gamma energies rise. This decrease was slower at higher energies. The dominance of Compton scattering also showed a smooth decrease in the second region [44,45]. However, a decrease against increasing gamma-ray energy was also reported for MAC values similar to LAC. The highest MAC values for the ASSPC sample were recorded at all incident photon energies. For example, MAC values were recorded as 25,246, 25,703, and 25,827 (cm^2^/g) for ASSC, ASBSC, and ASSPC samples at 15 MeV photon energy, respectively. The effect of high atomic number can explain this in the material composition in that ASSPC sample has the maximum Pb additive in its structure. The gamma shielding capability of shielding materials should also be tested in terms of the half-value layer (HVL) transmission factor.

**Figure 2:**
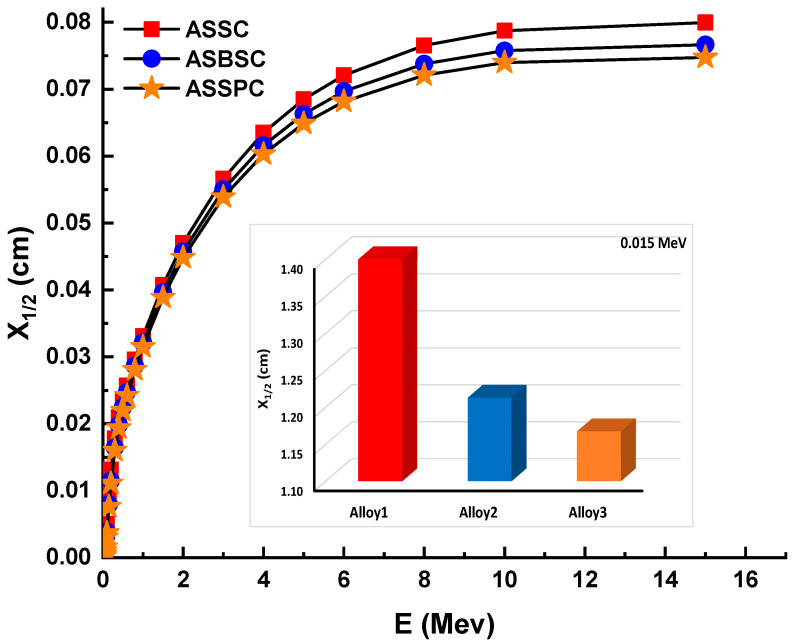
Half value layer (X_1/2_) for the 3 types of alloys.

**Figure 3:**
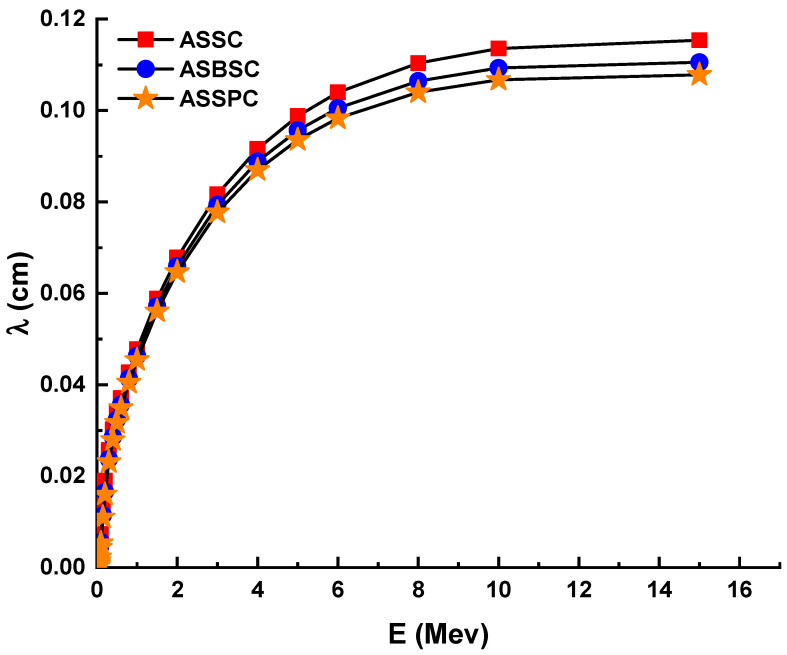
Mean free path (λ) for the 3 types of alloys.

The term HVL (also known as X_1/2_) is significant in radiation shielding research since it allows for the quantification of the material thickness required to halve the initial gamma-ray intensity. This is because radiation studies necessitate that shielding needs be determined in advance depending on the kind and energy of the radiation used. Therefore, the quantity of the HVL required for each kind of prospective shielding material should be determined in terms of a more complete understanding of the gamma ray attenuation capabilities during the incoming gamma ray’s contact with the attenuator specimen. Variation of HVL (cm) values of investigated glasses as a function of incident photon energy (MeV) is shown in Figure 2. As indicated in the Materials and Methods section, the linear attenuation coefficients and HVL quantities have an inverse relationship. As a result, it is reasonable to anticipate that the minimum HVL values would be found in the sample with the maximum linear attenuation coefficient values. Fortunately, our results indicated that the ASSPC sample with the highest alloy density and linear attenuation coefficients has the minimum HVL values. For example, HVLs were reported to be 0.033, 0.032, and 0.031 cm for ASSC, ASBSC, and ASSPC samples at 1 MeV photon energy, respectively. The term mean free path (MFP, λ) is the route taken by a photon through a substance without colliding. Therefore, it can be said that a lower distance in MFP values is a clear indicator for superior attenuation properties. Figure 3 depicts the variation of MFP (cm) values of investigated glasses as a function of incident photon energy (MeV). As mentioned above for previous attenuation properties, the ASSPC sample with the highest alloy density was also reported with its minimum MFP values. For instance, MFP values (cm) were reported as 0.048, 0.046, and 0.045 for ASSC, ASBSC, and ASSPC samples at 1 MeV photon energy, respectively.

Figure 3 illustrates the MFP values for the alloy samples examined. MFP values are often different than HVL values. Additionally, the lower MFP values for the ASSPC sample were claimed to be the lowest among the examined glasses. The effective atomic number (*Z_eff_*) is a helpful means for determining the suitability of a material for gamma applications. It is connected to the partial photon mitigation phase.

**Figure 4:**
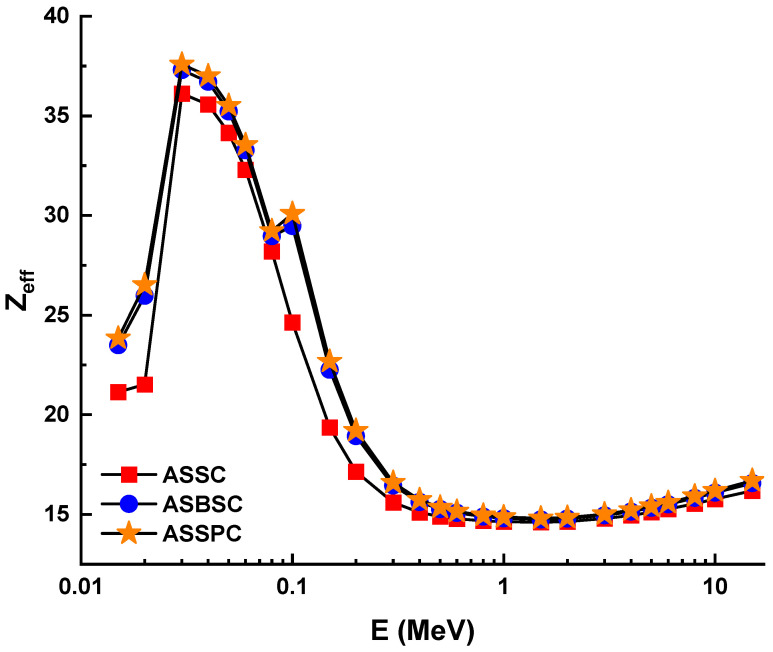
Effective atomic number (*Z_eff_*) for the 3 types of alloys.

**Figure 5:**
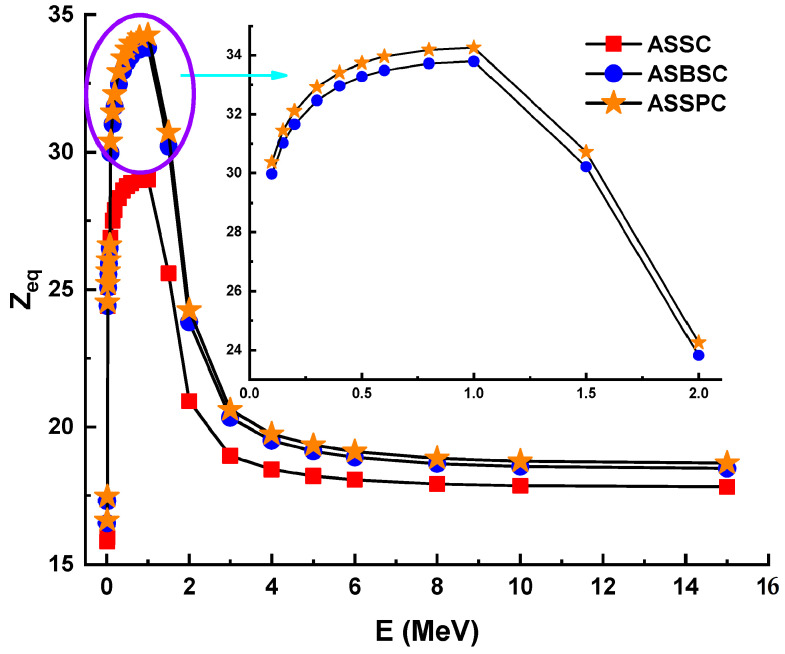
Equivalent atomic number (*Z_eq_*) for the 3 types of alloys.

Figure 4 illustrates the variation of the effective atomic number (*Z_eff_*) values of investigated alloys as a function of incident photon energy (MeV). From Figure 4, one can observe that the sample ASSPC has the maximum values of *Z_eff_*, while ASSC sample has the minimum values of *Z_eff_*. This is because with the increasing amount of Bi and Pb ions (*Z* = 83 and 82) and decreasing amount of Al ions (*Z* = 13), *Z_eff_* will be increased. We found that the maximum *Z_eff_* for the ASSPC sample was registered at 30 keV with a value of 37.58. Furthermore, the *Z_eff_* discontinuity at 0.1 MeV could be attributable to Bi and Pb k-absorption edges. On the other hand, equivalent atomic numbers (*Z_eq_*) for the three types of alloys were determined to calculate exposure buildup factor (EBF) and energy absorption buildup factor (EABF) using the geometry progressive (G-P) fitting method. The variation of *Z_eq_* is shown in Figure 5. As can be seen from the figure, a sharp increment was observed in the low-energy region. Next, a sharp decrease was reported until 2 MeV photon energy. *Z_eq_* values were observed as a maximum at 1 MeV photon energy. The numerical values of *Z_eq_* can be listed as 2900, 3380, and 3426 for ASSC, ASBSC, and ASSPC samples at 1 MeV, respectively.

Nevertheless, the greatest *Z_eq_* values were observed for all photon energy for the ASSPC sample. The buildup factor is a correction factor for the impact of dispersed radiation that takes into account any secondary particles in the medium. The accumulation variables must be considered to account for the accumulation of secondary ionizing radiation. Thus, the accumulation factor is a multiplier that compensates for the response to non-confronted photons, including the contribution of scattered photons. Therefore, the buildup factor is a multiplier that compensates for the response to non-confronted photons and incorporates the contribution of scattered photons. The term “buildup factor” has two sub-definitions: exposure buildup factor (EBF) and energy absorption buildup factor (EABF). Between 0.5 and 40 mfp, the EBF and EABF values of three different alloys were found using the G-P fitting technique.

**Figure 6:**
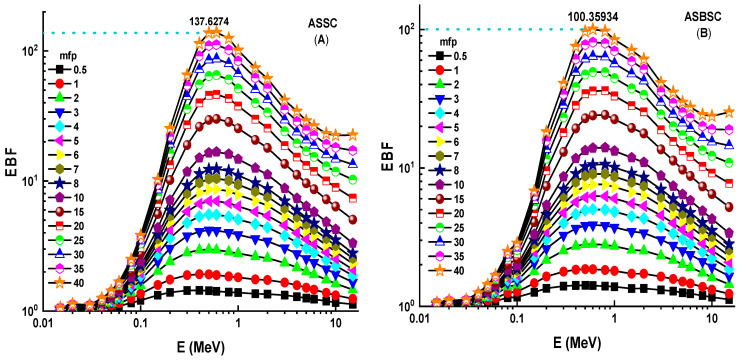

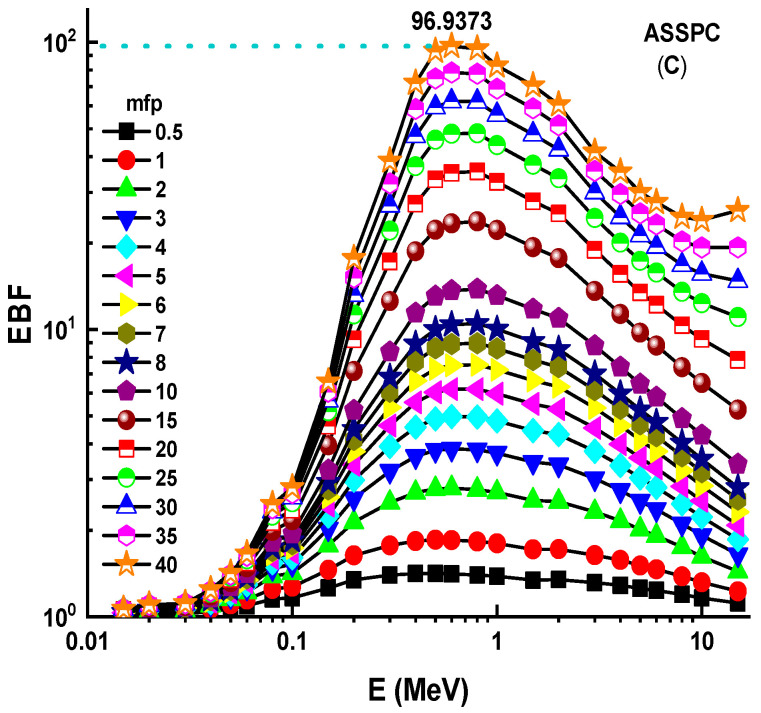
(**A**–**C**) Exposure buildup factor (EBF) for the 3 types of alloys.

**Figure 7:**
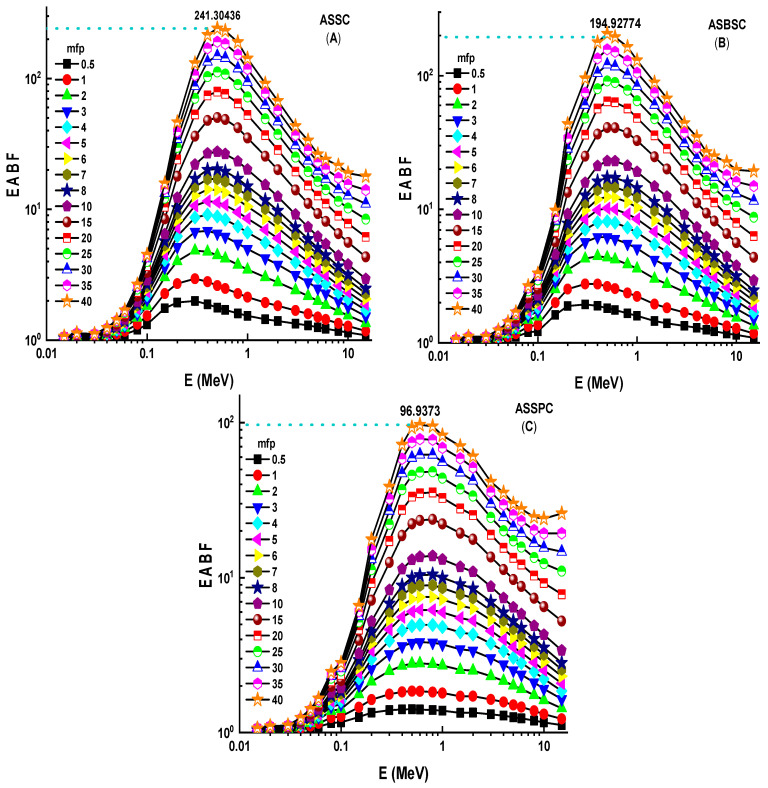
(**A**–**C**) Energy absorption buildup factor (EABF) for the 3 types of alloys.

**Figure 8:**
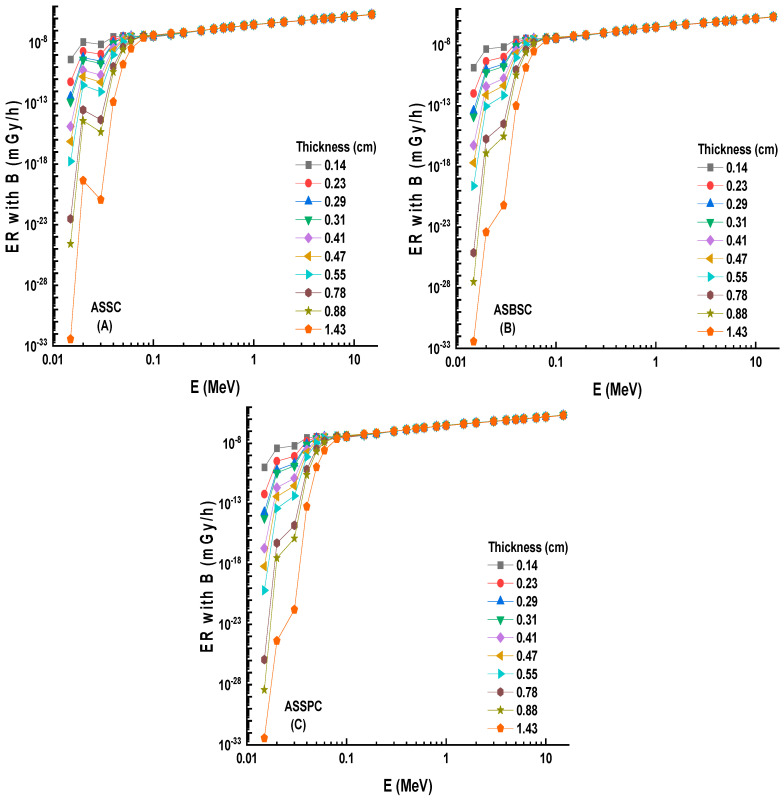
(**A**–**C**) Dependence of the exposure rate with buildup factor on thickness of 3 shields.

The fluctuation of EBF values as a function of energy (MeV) for various mfp values is shown in Figure 6A–C. These graphs demonstrate that the various penetration depths up to 40 mfp are composed of three distinct EBF vs. photon energy zones. These areas are essentially connected to photon–matter interactions. The first area revealed peaks due to the photoelectric phenomena occurring near the binding energy of the high atomic number elements. Subsequently, the Compton phenomena region saw almost consistent EBF values. Finally, the third zone is important for pair creation since the EBF increased somewhat due to absorption processes [18]. Among the investigated alloys, the Alloy3 sample showed the lowest EBF values. A numerical demonstration can be seen for 40 mfp penetration depths. At 40 mfp, the highest EBF values were reported as 1,376,274, 1,003,593, and 969,373 for ASSC, ASBSC, and ASSPC samples. This is a remarkable indicator of gamma-ray shielding capabilities of materials. Therefore, the ASSPC sample with the lowest EBF values can be underlined as the best sample among the investigated alloy specimens. A similar trend was also reported for EABF values, as shown in Figure 7A–C. At 40 mfp, the highest EBF values were reported as 2,413,043, 1,949,277, and 9,69,373 for ASSC, ASBSC, and ASSPC samples, respectively. It can be said that Alloy3 showed a strong consistency in shielding properties against gamma-rays in terms of EBF and EABF parameters. Another important shielding parameter, namely, exposure rate (ER), was also determined with and without buildup factor for ten different material thicknesses, such as 0.14, 0.23, 0.29, 0.31, 0.41, 0.47, 0.55, 0.78, 0.88, and 14.43 cm. Figure 8A–C shows the dependence of the exposure rate with buildup factor on the thickness of three different alloys, respectively. The dose point for measuring the exposure rate is directly behind the alloy, traced in a logarithmic scale, in order to widen the curve and maximize the difference between the values. As the energy rises, the rise in the exposure rate is seen as a general trend. Once again, ASSPC has the lowest exposure rate in all energies, while ASSC has the highest. For example, ERs of ASSC, ASBSC, and ASSPC samples with buildup were reported as 0.000002083, 0.000002075, and 0.00000207 for 1.43 cm at 15 MeV photon energy, respectively.

**Figure 9:**
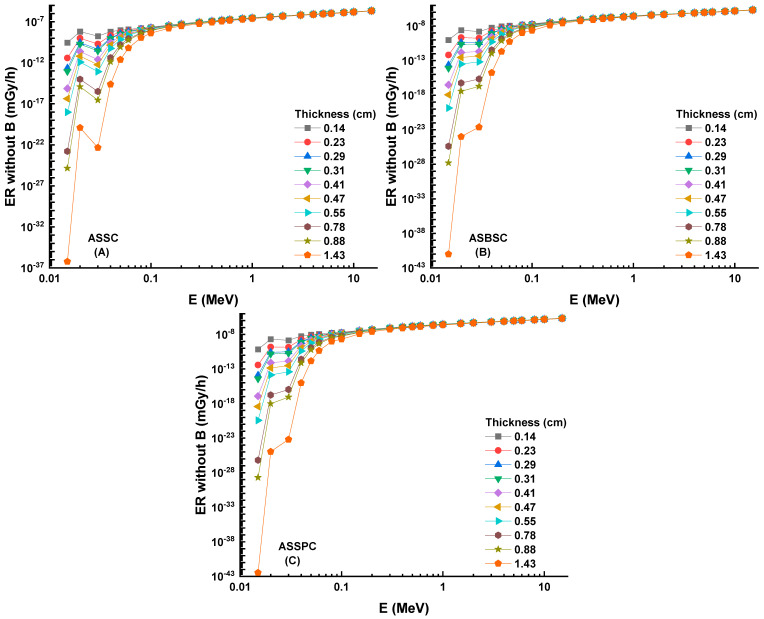
(**A**–**C**) Dependence of the exposure rate without buildup factor on the thickness of 3 shields.

**Figure 10:**
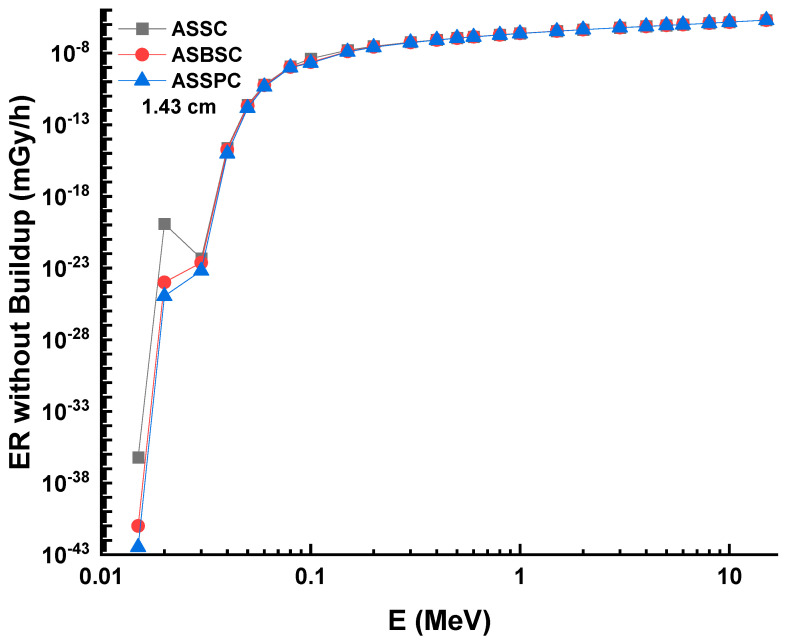
Exposure rate without buildup factor versus energy.

**Figure 11:**
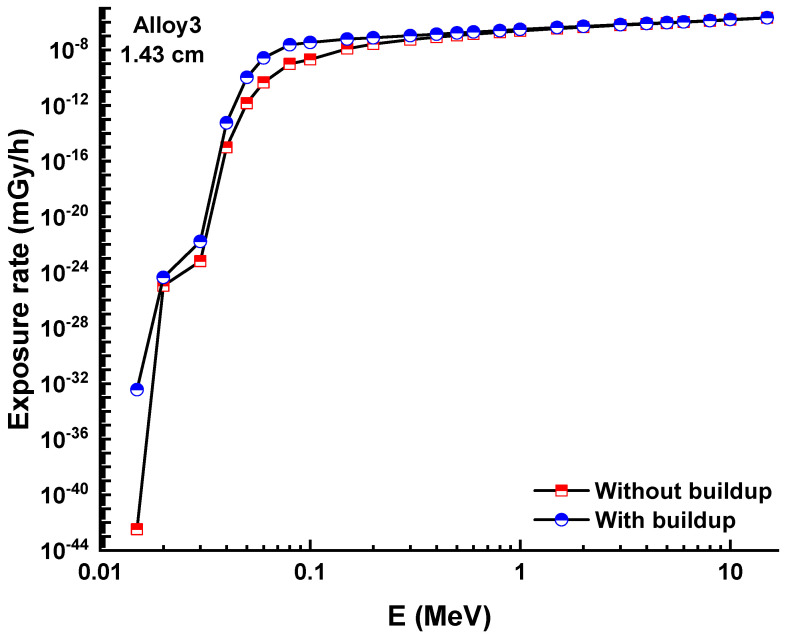
Exposure rate with and without buildup factor versus energy for Alloy3.

**Figure 12:**
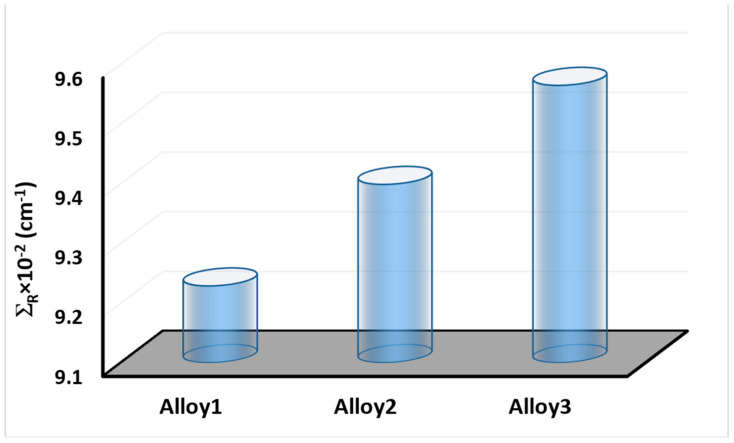
Fast neutron removal cross section (Σ*_R_*) for 3 alloys.

A similar situation was also reported for ERs without buildup factor, as shown in Figure 9A–C. Dependence of the exposure rate without buildup factor on the thickness of three shields. ERs of ASSC, ASBSC, and ASSPC samples without buildup were reported as 0.000002012, 0.000002001, and 0.000001995 for 1.43 cm at 15 MeV photon energy, respectively. A clear demonstration of exposure rate with/without buildup factor versus energy for 1.43 cm thickness can be seen in Figure 10 and Figure 11, respectively. Although the differences were reported with relative differences for ERs with/without buildup factor, the lowest values were recorded for ASSPC sample at all photon energies. Finally, Figure 12 shows the mass removal cross sections (Σ*_R_*) of alloy samples that were theoretically determined against fast neutrons. This parameter is a useful indicator for the attenuation properties of shielding materials against fast neutrons. The highest Σ*_R_* value was reported for ASSPC even though the numerical differences between the samples were not high. The contribution of Bi in the ASSPC sample is minimum. Therefore, elemental mass fraction, and accordingly, contribution of highest Z element (Bi = 83) is minimum. Therefore, it can be considered as an expected situation between the investigated alloy samples.

## 4. Conclusions

The word alloy refers to a material type that is well-known in the field of radiation equipment. Due to their improved material characteristics, alloys may be used directly for radiation shielding and beam collimation. The purpose of this research is to determine the relationship between dose rates and nuclear radiation shielding qualities in many alloy samples. At a variety of energy levels, the mass attenuation coefficients were determined. The mass attenuation coefficient values rose as the amount of Pb reinforcement increased in the samples: ASSC < ASBSC < ASSPC. HVL and MFP values are the lowest in the ASSPC sample. The findings indicate that substituting Pb for Bi in a recent alloy structure has a monotonic influence on several kinds of shielding properties. As a result, it can also be inferred that Pb is an exceptional tool for enhancing the shielding capabilities of the examined alloy structures. However, further research is required to identify other forms of additive materials that may be utilized in place of harmful Pb in alloy shields.

## Figures and Tables

**Table 1:** Elemental compositions (wt%) and density of alloys.

Code	Al	Sn	Bi	Si	Pb	Cu	g/cm^3^
ASSC	82	15		2.2		0.8	3.43
ASBSC	81.46	13.1	2.44	2.2		0.8	3.52
ASSPC	81.03	13.17		2.2	2.8	0.8	3.59

## Data Availability

The data presented in this study are available on request from the corresponding author.

## References

[1] Ogundare F.O., Olarinoye I.O., Obed R.I. (2009). Estimation of patients’ organ doses and conceptus doses from selected X-ray examinations in two Nigeria X-ray centres. Radiat. Prot. Dosim..

[2] Zakaly H.M., Rammah Y., Tekin H., Ene A., Badawi A., Issa S.A. (2022). Nuclear shielding performances of borate/sodium/potassium glasses doped with Sm^3+^ ions. J. Mater. Res. Technol..

[3] Rahman R.O.A., Hung Y.-T. (2019). Application of Ionizing Radiation in Wastewater Treatment: An Overview. Water.

[4] Zakaly H.M., Ashry A., El-Taher A., Abbady A.G., Allam E.A., El-Sharkawy R.M., Mahmoud M.E. (2021). Role of novel ternary nanocomposites polypropylene in nuclear radiation attenuation properties: In-depth simulation study. Radiat. Phys. Chem..

[5] Olarinoye I., Ogundare F. (2017). Optical and microstructural properties of neutron irradiated RF- sputtered amorphous alumina thin films. Optik.

[6] Ogundare F., Olarinoye I. (2016). He^+^ induced changes in the surface structure and optical properties of RF-sputtered amorphous alumina thin films. J. Non-Cryst. Solids.

[7] Henaish A.M.A., Mostafa M., Salem B.I., Zakaly H.M.H., Issa S.A.M., Weinstein I.A., Hemeda O.M. (2020). Spectral, electrical, magnetic and radiation shielding studies of Mg-doped Ni–Cu–Zn nanoferrites. J. Mater. Sci. Mater. Electron..

[8] Perişanoğlu U., El-Agawany F.I., Tekin H.O., Kavaz E., Zakaly H.M.H., Issa S.A.M., Zaid M.H.M., Sidek H.A.A., Matori K.A., Rammah Y.S. (2021). Multiple characterization of some glassy-alloys as photon and neutron shields: In-silico Monte Carlo investigation. Mater. Res. Express.

[9] Agar O., Sayyed M., Akman F., Tekin H.O., Kaçal M. (2019). An extensive investigation on gamma ray shielding features of Pd/Ag-based alloys. Nucl. Eng. Technol..

[10] Akman F., Sayyed M., Kaçal M., Tekin H. (2019). Investigation of photon shielding performances of some selected alloys by experimental data, theoretical and MCNPX code in the energy range of 81 keV–1333 keV. J. Alloys Compd..

[11] Kilic G., Ilik E., Issa S.A.M., Issa B., Al-Buriahi M.S., Issever U.G., Zakaly H.M.H., Tekin H.O. Ytterbium (III) oxide reinforced novel TeO_2_–B_2_O_3_–V_2_O_5_ glass system: Synthesis and optical, structural, physical and thermal properties. Ceram. Int..

[12] Rashad M., Saudi H.A., Zakaly H.M.H., Issa S.A.M., Abd-Elnaiem A.M. (2021). Control optical characterizations of Ta^+5^–doped B_2_O_3_–Si_2_O–CaO–BaO glasses by irradiation dose. Opt. Mater. (Amst)..

[13] Akman F., Kaçal M., Sayyed M., Karataş H. (2019). Study of gamma radiation attenuation properties of some selected ternary alloys. J. Alloys Compd..

[14] Kaur S., Kaur A., Singh P.S., Singh T. (2016). Scope of Pb-Sn binary alloys as gamma rays shielding material. Prog. Nucl. Energy.

[15] Singh J., Singh H., Sharma J., Singh T., Singh P.S. (2018). Fusible alloys: A potential candidate for gamma rays shield design. Prog. Nucl. Energy.

[16] Singh T., Kaur A., Sharma J., Singh P.S. (2018). Gamma rays’ shielding parameters for some Pb-Cu binary alloys. Eng. Sci. Technol. Int. J..

[17] Aygün B., Şakar E., Korkut T., Sayyed M., Karabulut A., Zaid M. (2019). Fabrication of Ni, Cr, W reinforced new high alloyed stainless steels for radiation shielding applications. Results Phys..

[18] Chen X., Liu L., Liu J., Pan F. (2015). Microstructure, electromagnetic shielding effectiveness and mechanical properties of Mg–Zn–Y–Zr alloys. Mater. Des..

[19] Singh V.P., Badiger N.M. (2016). An investigation on gamma and neutron shielding efficiency of lead-free compounds and alloys. Indian. J. Pure Appl. Phys..

[20] Ali A.M., Issa S.A., Ahmed M.R., Saddeek Y.B., Zaid M.H.M., Sayed M., Somaily H.H., Tekin H.O., Sidek H.A.A., Matori K.A. (2020). Promising applicable heterometallic Al_2_O_3_/PbO_2_ nanoparticles in shielding properties. J. Mater. Res. Technol..

[21] El-Denglawey A., Issa S.A.M., Saddeek Y.B., Tekin H.O., Zakaly H.M.H. (2021). The Impact of PbF_2_-Based Glasses on Radiation Shielding and Mechanical Concepts: An Extensive Theoretical and Monte Carlo Simulation Study. J. Inorg. Organomet. Polym. Mater..

[22] Alzahrani J.S., Alrowaili Z., Saleh H., Hammoud A., Alomairy S., Sriwunkum C., Al-Buriahi M. (2021). Synthesis, physical and nuclear shielding properties of novel Pb–Al alloys. Prog. Nucl. Energy.

[23] Sathish K., Manjunatha H., Seenappa L., Sridhar K., Sowmya N., Raj S.A.C. (2022). Gamma, X-ray and neutron shielding properties of iron boron alloys. Mater. Today Proc..

[24] Waly E.-S.A., Al-Qous G.S., Bourham M.A. (2018). Shielding properties of glasses with different heavy elements additives for radiation shielding in the energy range 15–300 keV. Radiat. Phys. Chem..

[25] Rashad M., Tekin H., Zakaly H.M., Pyshkina M., Issa S.A., Susoy G. (2020). Physical and nuclear shielding properties of newly synthesized magnesium oxide and zinc oxide nanoparticles. Nucl. Eng. Technol..

[26] El-Taher A., Zakaly H.M.H., Pyshkina M., Allam E.A., El-Sharkawy R.M., Mahmoud M.E., Abdel-Rahman M.A.E. (2021). A comparative Study Between Fluka and Microshield Modeling Calculations to study the Radiation-Shielding of Nanoparticles and Plastic Waste composites. Z. Anorg. Allg. Chem..

[27] Şakar E., Özpolat Ö.F., Alım B., Sayyed M., Kurudirek M. (2020). Phy-X/PSD: Development of a user friendly online software for calculation of parameters relevant to radiation shielding and dosimetry. Radiat. Phys. Chem..

[28] Wang Z.-M., Yang Q., Sun Z.-P., Zhang B.-R., Zhao W., Rao W.-F. (2020). The effects of Bi and Pb on the soft phase in Al_82_Sn_15_Si_2.2_Cu_0.8_ sliding bearing alloy. Mater. Charact..

[29] MicroShield Radiation Software|Grove Software. https://radiationsoftware.com/microshield.

[30] Saudi H., Issa S.A., Elazaka A., Zakaly H.M., Kilic G., Tekin H. (2021). Exploration of material characteristics of tantalum borosilicate glasses by experimental, simulation, and theoretical methods. J. Phys. Chem. Solids.

[31] Zakaly H.M.H., Ene A., Olarinoye O.I., Marzouk S.Y., Abdel-Hafez S.H., Shams M.S., Rammah Y.S. (2021). Investigation of Er^3+^ Ions Reinforced Zinc-Phosphate Glasses for Ionizing Radiation Shielding Applications. Materials.

[32] Tekin H.O., Bilal G., Zakaly H.M.H., Kilic G., Issa S.A.M., Ahmed E.M., Rammah Y.S., Ene A. (2021). Newly Developed Vanadium-Based Glasses and Their Potential for Nuclear Radiation Shielding Aims: A Monte Carlo Study on Gamma Ray Attenuation Parameters. Materials.

[33] Zakaly H.M., Saudi H., Tekin H., Rashad M., Issa S.A., Rammah Y., Elazaka A., Hessien M., Ene A. (2021). Glass fabrication using ceramic and porcelain recycled waste and lithium niobate: Physical, structural, optical and nuclear radiation attenuation properties. J. Mater. Res. Technol..

[34] Gerward L., Guilbert N., Jensen K., Levring H. (2004). WinXCom—A program for calculating X-ray attenuation coefficients. Radiat. Phys. Chem..

[35] Almisned G., Tekin H.O., Bilal G., Ene A., Kilic G., Issa S.A.M., Algethami M., Zakaly H.M.H. (2021). Trivalent Ions and Their Impacts on Effective Conductivity at 300 K and Radio-Protective Behaviors of Bismo-Borate Glasses: A Comparative Investigation for Al, Y, Nd, Sm, Eu. Materials.

[36] Zakaly H.M., Issa S.A., Tekin H., Badawi A., Saudi H., Henaish A., Rammah Y. (2022). An experimental evaluation of CdO/PbO-B_2_O_3_ glasses containing neodymium oxide: Structure, electrical conductivity, and gamma-ray resistance. Mater. Res. Bull..

[37] (1990). Gamma Ray Attenuation Coefficient and Buildup Factors for Engineering.

[38] Kavaz E., Yorgun N.Y. (2018). Gamma ray buildup factors of lithium borate glasses doped with minerals. J. Alloys Compd..

[39] Tekin H., Kilicoglu O. (2020). The influence of gallium (Ga) additive on nuclear radiation shielding effectiveness of Pd/Mn binary alloys. J. Alloys Compd..

[40] Tekin H.O., Issa S.A.M., Kavaz E., Guclu E.E.A. (2019). The direct effect of Er_2_O_3_ on bismuth barium telluro borate glasses for nuclear security applications. Mater. Res. Express.

[41] Issa S.A., Ahmad M., Tekin H., Saddeek Y., Sayyed M. (2019). Effect of Bi_2_O_3_ content on mechanical and nuclear radiation shielding properties of Bi_2_O_3_-MoO_3_-B_2_O_3_-SiO_2_-Na_2_O-Fe_2_O_3_ glass system. Results Phys..

[42] Issa S.A., Darwish A., El-Nahass M. (2017). The evolution of gamma-rays sensing properties of pure and doped phthalocyanine. Prog. Nucl. Energy.

[43] Zakaly H.M.H., Saudi H.A., Issa S.A.M., Rashad M., Elazaka A.I., Tekin H.O., Saddeek Y.B. (2021). Alteration of optical, structural, mechanical durability and nuclear radiation attenuation properties of barium borosilicate glasses through BaO reinforcement: Experimental and numerical analyses. Ceram. Int..

[44] Uosif M., Mostafa A., Issa S.A., Tekin H., Alrowaili Z., Kilicoglu O. (2020). Structural, mechanical and radiation shielding properties of newly developed tungsten lithium borate glasses: An experimental study. J. Non-Cryst. Solids.

[45] Alatawi A., Alsharari A.M., Issa S.A., Rashad M., Darwish A., Saddeek Y., Tekin H. (2020). Improvement of mechanical properties and radiation shielding performance of AlBiBO_3_ glasses using yttria: An experimental investigation. Ceram. Int..

